# A framework for detecting unfolding emergencies using humans as sensors

**DOI:** 10.1186/s40064-016-1674-y

**Published:** 2016-01-19

**Authors:** Marco Avvenuti, Mario G. C. A. Cimino, Stefano Cresci, Andrea Marchetti, Maurizio Tesconi

**Affiliations:** Department of Information Engineering, University of Pisa, Largo L. Lazzarino 1, 56122 Pisa, Italy; Bell Labs, Alcatel-Lucent, Route de Villejust, 91620 Nozay, Paris, France; Institute of Informatics and Telematics (IIT), National Research Council (CNR), Via G. Moruzzi 1, 56124 Pisa, Italy

**Keywords:** Twitter, Social sensing, Social media mining, Event detection, Crisis informatics, Emergency management

## Abstract

The advent of online social networks (OSNs) paired with the ubiquitous proliferation of smartphones have enabled social sensing systems. In the last few years, the aptitude of humans to spontaneously collect and timely share context information has been exploited for emergency detection and crisis management. Apart from event-specific features, these systems share technical approaches and architectural solutions to address the issues with capturing, filtering and extracting meaningful information from data posted to OSNs by networks of human sensors. This paper proposes a conceptual and architectural framework for the design of emergency detection systems based on the “human as a sensor” (HaaS) paradigm. An ontology for the HaaS paradigm in the context of emergency detection is defined. Then, a modular architecture, independent of a specific emergency type, is designed. The proposed architecture is demonstrated by an implemented application for detecting earthquakes via Twitter. Validation and experimental results based on messages posted during earthquakes occurred in Italy are reported.

## Background

Established public safety systems are based on centralized emergency detection approaches, often relying on expensive infrastructures of physical sensors which may not be available everywhere. The proliferation of handheld devices, equipped with a large number of sensors and communication capabilities, can significantly extend, or possibly substitute, conventional sensing by enabling the collection of data through networks of humans. Novel paradigms such as crowd-, urban- or citizen-sensing have been coined to describe how information can be sourced from the average individual in a coordinated way. Data gathering can be either participatory or opportunistic, depending on whether the user intentionally contributes to the acquisition campaign (possibly receiving an incentive), or she simply acts as the bearer of a sensing device from which data is transparently collected by some situation-aware system (Sheth 2009; Kapadia et al. 2009; Cimino et al. 2012).

In this scenario, the advent of online social network (OSN) platforms, such as Twitter, Weibo and Instagram, that have grown bigger becoming a primary hub for public expression and interaction, has added facilities for ubiquitous and real-time data-sharing (Demirbas et al. 2010). These unprecedented sensing and sharing opportunities have enabled situations where individuals not only play the role of sensor operators, but also act as data sources themselves. In fact, humans have a great aptitude in processing and filtering observations from their surroundings and, with communication facilities at hand, in readily sharing the information they collect (Srivastava et al. 2012). This spontaneous behavior has driven a new challenging research field, called “social sensing” (Aggarwal and Abdelzaher 2013), investigating how human-sourced data, modeled by the “human as a sensor” (HaaS) paradigm (Wang et al. 2014), can be gathered and used to gain situational awareness and to nowcast events (Lampos and Cristianini 2012) in different domains such as health, transportation, energy, social and political crisis, and even warfare. Among the advantages of social sensing is the natural tendency of OSN users to promptly convey information about the context (Liang et al. 2013; Cresci et al. 2015b) and that those proactively posted messages, especially when witnessing emergency situations, are likely to be free of pressure or influence (Zhou et al. 2012). The utmost case is Twitter, where users are encouraged to make their messages (*tweets*) publicly available by default and where, due to the 140 characters length limitation, they are forced to share more topic-specific content.

Given this picture, it is not surprising that OSNs, and Twitter in particular, have drawn the attention of designers of decision support systems for emergency management, and that during recent disasters, such as the Tōhoku earthquake and tsunami (Japan—2011), the Hurricane Sandy (Central and North America—2012) and the Himalayan earthquake (Nepal—2015), civil protection agencies turned to the Web and to OSN data to help tracking stricken locations, assessing the damage and coordinating the rescue efforts. Based on the observation that an unfolding emergency is likely to give rise to a burst of alerting messages, which may be used to early detect the event, followed by more reflective messages, whose content may be used to understand its consequences, several systems have focused on the collection and analysis of messages shared in areas affected by disasters (Hughes and Palen 2009; Bagrow et al. 2011; Adam et al. 2012; Gao et al. 2014; Avvenuti et al. 2014a. However, such information is often unstructured, heterogeneous and fragmented over a large number of messages in such a way that it cannot be directly used. It is therefore mandatory to turn that messy data into a number of clear and concise messages for emergency responders (Cresci et al. 2015b). Challenging issues highlighted and faced by pioneer systems include the real-time acquisition of unstructured data not specifically targeted to the system (data is often free text without structure or codified semantics) (Goolsby 2010), the extraction of critical data overwhelmed by high flood of meaningless babbles, the identification of the most stricken areas in the aftermath of an emergency (Cresci et al. 2015c; Sakai and Tamura 2015), security and privacy issues including the lack of guarantee that human sensors correctly deliver information about specific facts at specific times (Rosi et al. 2011).

Despite these common findings, an analysis of the state-of-the-art in the field of social sensing-based emergency management systems highlights a multitude of domain-specific, unstructured and heterogeneous solutions. In fact, in the literature the design of monolithic and vertical ad-hoc solutions still prevails over architectural approaches addressing modularity, generality and flexibility (Imran et al. 2015). This paper presents a framework for detecting emergent crisis events using humans as sensors. According to the framework, different emergency types (e.g., seismic, hydrological, meteorological) can be detected by configuring a software architecture, where re-usable components can adapt to different contents and patterns of messages posted to the OSN while the event unfolds. The contribution of the paper is both conceptual and practical. To the purpose of deepening and sharing the understanding of the properties and relationships of data provided by human sensors, we have defined a terminology and an ontology for the HaaS paradigm in the context of emergency detection. From the practical point of view, we have designed a domain-independent, architectural and modular framework that encompasses the vast majority of systems proposed to date. The effectiveness of the proposed architecture in solving common problems, such as data capturing, data filtering and emergency event detection, has been demonstrated by a proof-of-concept implementation involving earthquake detection via Twitter. The application has been validated using datasets of tweets collected during earthquakes occurred in Italy.

## Related work

In this section, we outline the most relevant works in the field, discussing the main differences with our approach as well as the main similarities, in order to point out the works that inspired our architectural model. Thus, this section corroborates our approach under the more general umbrella of the HaaS paradigm for emergency management.

Several initiatives, both in scientific and in application environments, have been developed in the last few years with the aim of exploiting information available on social media during emergencies. Works proposed in the literature either describe working systems employing solutions for some of the fundamental challenges of emergency management, or focus on a single specific challenge and thoroughly study it. The systems surveyed in this section present different degrees of maturity. Some have been deployed and tested in real-life scenarios, while others remain under development (Imran et al. 2015). The vast majority of these systems share goals or functionalities with the framework we are proposing and can be mapped, totally or in part, on the architecture subsequently defined. Among the proposed systems some approaches are tailored to suit requirements of a specific kind of emergency and are therefore domain-specific. Overall, many of the surveyed works present shortcomings regarding their reusability.

The works presented in Bartoli et al. (2015) and Foresti et al. (2015) describe novel emergency management platforms for smart public safety and situational awareness. The proposed solutions exploit both wireless sensor networks and social media to support decision-makers during crises. In Bartoli et al. (2015) a high-level framework is proposed which includes subsystems designed for the acquisition and the analysis of heterogeneous data. The subsystems working on social media data perform the data acquisition and data analysis tasks and can be directly mapped to the corresponding components of our architecture. In this framework data acquisition from social media has a marginal impact since it is activated only after the detection of an emergency. Thus Bartoli et al. (2015) only marginally deals with the challenges related to the acquisition and handling of a big stream of social media data. An example of an application scenario for the system is also proposed for hydrological risks such as floods and landslides. The ASyEM system (Foresti et al. 2015) focuses on data acquisition and data fusion. Authors introduce an offline methodology for the extraction of emergency-specific terms which are subsequently used by the online system to gather relevant messages from social media sources. The detection of an emergency is performed by means of a neural tree network previously trained during the offline phase. Both Bartoli et al. (2015) and Foresti et al. (2015) lack a data filtering component. Similarly to Foresti et al. (2015), the work discussed in Salfinger et al. (2015) employs data fusion techniques in a system designed to increase situational awareness during emergencies. Authors propose a high-level architecture for an adaptive framework exploiting both traditionally sensed data as well as social media data.

Among the various kinds of emergencies, seismic events are those which have been investigated the most in the last few years. Earthquake emergency management is a topic worth studying not only for the big threat seismic events pose on communities and infrastructures. The detailed earthquake characterization obtainable from seismographic networks can be exploited as a baseline for novel social media-based emergency management systems and leveraged to achieve better results in terms of responsiveness and situational awareness. The opportunities granted by the application of the HaaS paradigm to earthquake detection and response have been firstly envisioned in works such as Earle (2010), Allen (2012), and Crooks et al. (2013).

The study described in Sakaki et al. (2010, 2013) is one among the first works proposing techniques for emergency management based on social media data. Authors investigate the design and development of a social alert detection and earthquake reporting system. The detection of an event is performed by means of a bayesian statistical model. Authors carried out experiments to assess the quality of the detections and their responsiveness. Detection results are evaluated only by means of the Recall metric (ratio of correctly detected earthquakes among the total occurred earthquakes) and the system was able to timely detect 67.9 % (53 out of 78) of the earthquakes with JMA (Japan Meteorological Agency) scale 2 or more which occurred over 2 months. It is worth noting that the JMA scale can not be directly mapped into the worldwide-adopted Richter magnitude scale used in Table 1 to evaluate our system^1^. The approach proposed in Sakaki et al. (2010, 2013) is tested on both earthquakes and tornadoes and the achieved results seem convincing towards the employment of this solution for other large-scale emergencies as well. However, the work only focuses on the event detection task, without dealing with the definition of a full working system. Moreover, data acquisition is performed by means of the Twitter Search API^2^ which accesses to only a portion of the amount of tweets produced. While this limitation can be negligible for large scale events, it can impair the system’s ability to detect events felt by a small number of social sensors, thus limiting the reusability of this system for small-scale emergencies such as landslips, traffic jams, car accidents, etc.

US Geological Survey (USGS) efforts towards the development of an earthquake detection system based solely on Twitter data are described in Earle et al. (2012). The solution is evaluated with different settings according to the sensitivity of the event detection module. However, even in its best configuration, the system could only detect 48 globally distributed earthquakes out of the 5175 earthquakes occurred during the same time window. Also this system acquires data via the Twitter Search API, thus suffering from the same limitations described above. Basic data filtering concerns are taken into account and relevant messages are selected with a heuristic approach. Event detection is performed by a STA/LTA (short-term average/long-term average) algorithm. Although representing an interesting demonstration of the possibility to perform emergency event detection via social media, this system has a few shortcomings which severely limit its performances. The deeper level of analysis supported in our proposed architecture and performed in our implementation allow us to outperform USGS’s system. Overall, we believe the main reasons for our better performances lie in the adoption of more sophisticated filtering techniques (i.e. machine learning classifiers instead of heuristics) and a more powerful event detection algorithm (i.e. a burst detection algorithm instead of a STA/LTA). USGS kept on working on the project and recently announced the official employment of a Twitter earthquake detection system named TED (Tweet Earthquake Dispatch). As claimed by USGS, such detection system proved more responsive than those based on seismographs in regions where the number of seismographic stations is low^3^^,^^4^.

In Avvenuti et al. (2014a, b, 2015) is described the development of the Earthquake Alert and Report System (EARS). EARS is a real-time platform designed for the detection and the assessment of the consequences of earthquakes from social media data. The proposed solution employs data mining and natural language processing techniques to enhance situational awareness after seismic events. Although the proposed system is domain-specific and employed only in the field of earthquake emergency management, the discussion in Avvenuti et al. (2014b) addresses issues common to all social media emergency management systems. Preliminary results of the works proposed in Sakaki et al. (2010, 2013); Earle et al. 2012) and Avvenuti et al. (2014a, b, 2015) are overall encouraging, especially in relation to the responsiveness of the detections. In the present work we built on the key features of these systems in order to design a solution applicable to a broad range of emergencies.

Situational awareness during emergencies is the goal of the work described in Yin et al. (2012). The Emergency Situation Awareness (ESA) platform operates over the Twitter stream by comparing terms used in recent tweets with those of a baseline. The baseline has been generated in an offline phase and represents a statistical model of the terms used during a fixed time window of several months. ESA raises alerts for every term which appears in recent tweets significantly more than in the baseline. The drawback of this approach is that the baseline does not account for topic seasonality. Moreover ESA does not perform data filtering neither employs keywords for the data acquisition and therefore many of the generated alerts are of little interest. ESA represents however one of the first domain-independent approaches to the problem of emergency management from social media. The core of the general ESA platform has been later expanded with ad-hoc filters and tailored to perform event detection in the earthquakes (Robinson et al. 2013) and wildfires (Power et al. 2013) domains. Other works have instead investigated the exploitation of social sensors for the detection of traffic jams (D’Andrea et al. 2015).

Crowdsourced crisis mapping from Twitter data is the goal of the systems proposed in Middleton et al. (2014), Cresci et al. (2015c). Crisis mapping concerns with the capturing, processing and display of data during a crisis with the goal of increasing situational awareness. Following an approach adopted in other previously reviewed works, these systems are composed of both offline and real-time (online) subsystems. The offline subsystems calculate baseline statistics during a historical period when no disasters occurred. Among the real-time subsystems Middleton et al. (2014) also includes a data filtering component which, similarly to Earle et al. (2012), applies heuristic rules to select relevant tweets. On the contrary, Cresci et al. (2015c) uses machine learning techniques to filter and analyze data.

Lastly, the study in Imran et al. (2015) presents a survey on computational techniques for social media data processing during emergencies and can be considered as a further reference for works in the fields of social media emergency management, crisis informatics and crisis mapping.

## Core concepts and functionalities

**Fig. 1:**
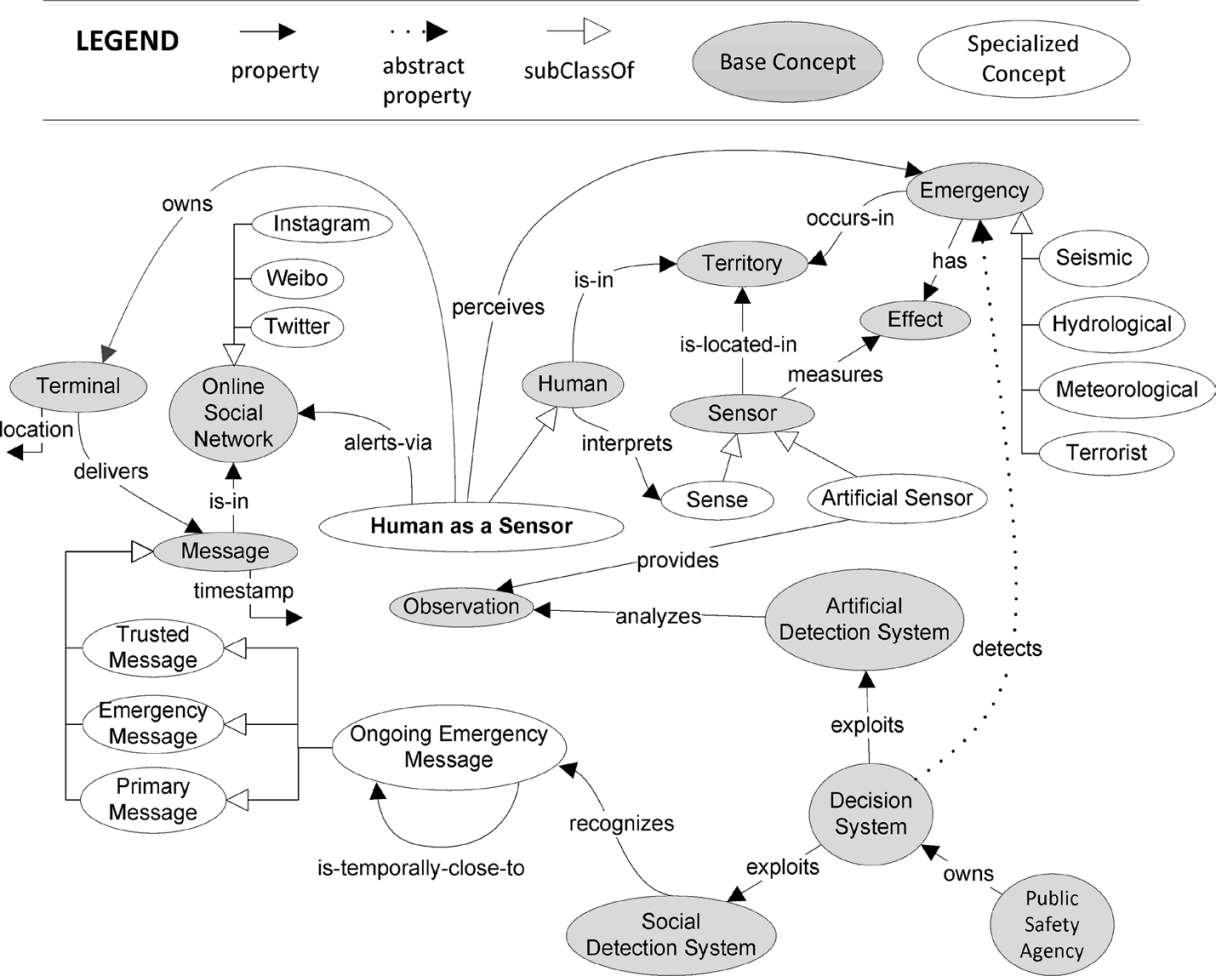
An ontological view of the HaaS paradigm for emergency management

Our conceptual framework is intended to operate in a broad class of domains. For this reason it should evolve from an explicit formal specification of terms and of relationships among them. This way, experts are supported with shared understanding of their domains of interest. A good specification serves as a basis to communicate in development, to guarantee consistency, to minimize misunderstanding and missed information, to overcome barriers to the acquisition of specifications, to reuse and analyze domain knowledge, and to separate it from operational knowledge. Among the suitable formalisms, ontologies are structured vocabularies with definitions of basic concepts and relations among them. Ontologies have interesting properties that can be formally verified, such as completeness, correctness, consistency, and unambiguity (Siegemund et al. 2011).

In this section we introduce the terminology of the “human as a sensor” (HaaS) paradigm via an ontology diagram. In Fig. 1 base concepts are enclosed in gray ovals and connected by properties, represented by black directed edges. The fundamental property is on the right: *Decision System detects Emergency*. This property cannot be directly sensed (i.e., instantiated) by the system, and is therefore represented as an *abstract* property, shown by a dotted edge. Indeed the overall decision system is aimed at indirectly detecting emergencies by means of a series of information provided by sensors. As the system should be scalable in terms of types of emergency, different specific emergencies have been considered. In figure, *Seismic*, *Hydrological*, *Meteorological*, and *Terrorist* are examples of specialized concepts, shown with white ovals and connected by white directed edges to the base concept.

A *Decision System* is *owned by a Public Safety Agency*, and *exploits* both *Artificial* and *Social Detection Systems*. The former is a conventional system based on physical sensors: an *Artificial Detection System analyzes Observations*, which are *provided by Artificial Sensors*, i.e., a type of specialized *Sensor*. Another type of specialized sensor is human *Sense*, which is interpreted by *Humans*. Here, the concept *Human acts as a Sensor* can then be derived as a specialized human. Indeed, both *Human* and *Sensor* are in the *Territory*, where *Emergency occurs* and *Effects* of it are *measured by Sensors*. Differently from an artificial sensor, a *Human as a Sensor* is able to directly *perceive* an emergency and *owns a Terminal to deliver Messages in an Online Social Network*. For this reason, he can *alert via an Online Social Network*. *Location* is a structural property of a terminal. Specialized examples of Online Social Networks are *Twitter*, *Weibo*, and *Instagram*.

**Fig. 2:**
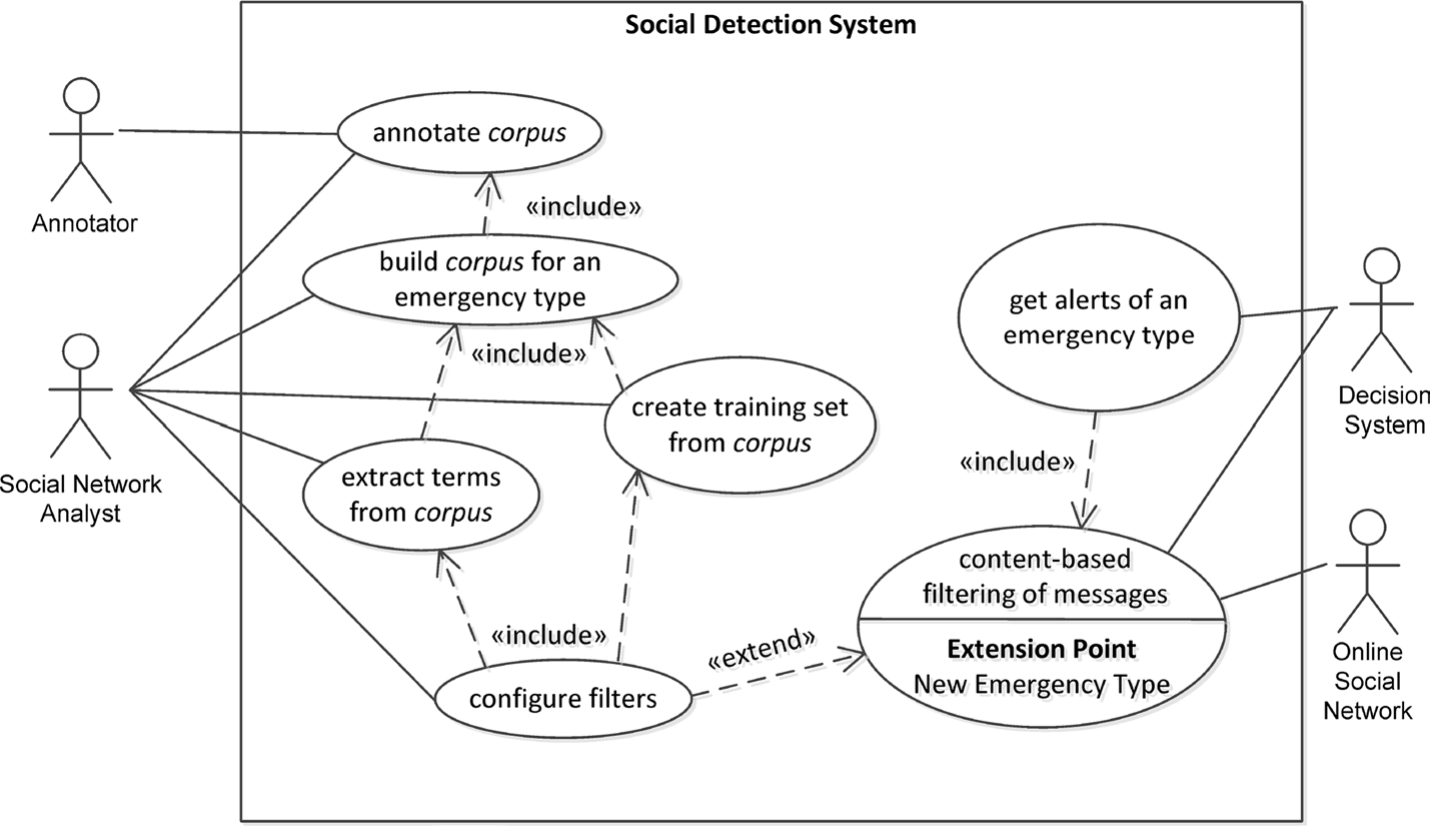
Use cases of the HaaS paradigm for emergency management

In the context of online detection, a structural property of a message is the *timestamp*. Other properties are content-based and must be recognized as specialized types: a *Trusted Message*, i.e., a message which is not sent for malicious, disruptive or abusive purposes (Mendoza et al. 2010; Castillo et al. 2011); a *Primary Message*, i.e., a message sent by a user who is actually present at the referred event and can directly describe it (Kumar et al. 2013; Morstatter et al. 2014); an *Emergency Message*, i.e., a message reporting an actual social emergency and not, for instance, reporting a personal problem via an idiom made of emergency words (Avvenuti et al. 2014a). If all these properties are available in a single message, that message can be considered an instance of a further specialized concept, the *Ongoing Emergency Message*, which is a message reporting an ongoing emergency. In addition, an Ongoing Emergency Message must have another property: being *temporally close to* another message of the same typology. This way, the *Social Detection System recognizes* a number of temporally close messages. Thus, the detection of an actual social emergency encompasses many messages, differently arranged in time depending on the type of emergency.

Managing a *Social Detection System* requires interaction between different external agents (people or systems), represented in Fig. 2 as UML use cases. Here, interacting agents are called actors and are represented by the “stick man” icon, whereas functionalities available to actors are represented by an oval shape. An actor can communicate with the system through an association to a functionality, represented as a link. Use cases have been related to other use cases by the *extend* and *include* relationships, allowing to increment a use case and to specify a piece of the use case in a modular way, respectively. A relationship is represented as a dashed directed arrow, whose direction denotes dependency.

More specifically, for a given emergency type (e.g., earthquake, flooding, or their subtypes) the *Decision System* asks the *Social Detection System* (hereafter called System for the sake of brevity) to be prepared to *get alerts of that emergency type*. This functionality includes the activation of the *content-based filtering of messages*, which is in charge of providing, among the messages captured from the *Online Social Network* actor (e.g., Twitter), only those containing information related to the unfolding emergency situation. We call this use case the *online process*.

Emergency-specific knowledge of the content of messages is thus necessary to extend the System’s capability in recognising multiple emergency types. Such a knowledge can be extracted from a *message corpus*, a large and structured set of messages (electronically stored and processed), used for statistical analysis and hypothesis testing, checking occurrences or validating filtering within a specific emergency type. Extracted knowledge can be encoded as: (1) *terms* that are frequently contained in the target messages, established via statistical methods; (2) *features* extracted from a *training set* of target messages, established via machine learning methods; (3) parameters of collections of messages related to the same emergency event, established via statistical methods.

Thus, when a new emergency type has to be managed, the *content-based filtering of messages* functionality must be previously extended with emergency-specific knowledge provided by the *configure filters* functionality. This process is managed by the actor responsible for the System’s maintenance and configuration, the *Social Network Analyst*. Configuring filters includes *creating training sets and extracting terms from corpus*. *To build a corpus* includes to *annotate corpus*, in collaboration with a number of *Annotators*. We call the *configure filters* use case the *offline process*.

## Architectural design

The “human as a sensor” (HaaS) paradigm for emergency management so far determined has been used as a reference for designing an efficient, flexible and scalable software architecture. The analysis conducted in the previous section, as well as the findings reported in previous works, highlighted the fundamental challenges related to processing social media data for the detection of unfolding emergency situations (Imran et al. 2015). Such challenges comprehend: (1) data capturing, (2) data filtering and (3) emergency event detection. The challenge related to data capturing lies in gathering, among the sheer amount of social media messages, the most complete and specific set of messages for the detection of a given type of emergency. However, not all collected messages are actually related to an unfolding emergency, hence the need of a data filtering step to further reduce the noise among collected messages and retain only the relevant ones. Finally, techniques are needed in order to analyze relevant messages and infer the occurrence of an emergency event. The general framework for emergency management that we are proposing efficiently deals with all these aspects.

In this section the system logic is represented by a number of components and actors. A component represents a modular piece of logic whose external behavior can be concisely described to offer a platform-independent view. Each component may be developed in any programming language and by using one or more classes or procedures, since its internal algorithmic implementation is not detailed. Indeed, each component in the model can be replaced by another component supporting the same interfaces, thus providing modularity. Each actor represents a role played by a user interacting with the system components. Subsequently, a behavioral description of the system within its life cycle is also provided by means of a sequence of exchange messages between actors and components.

### Static view of the logical architecture

**Fig. 3:**
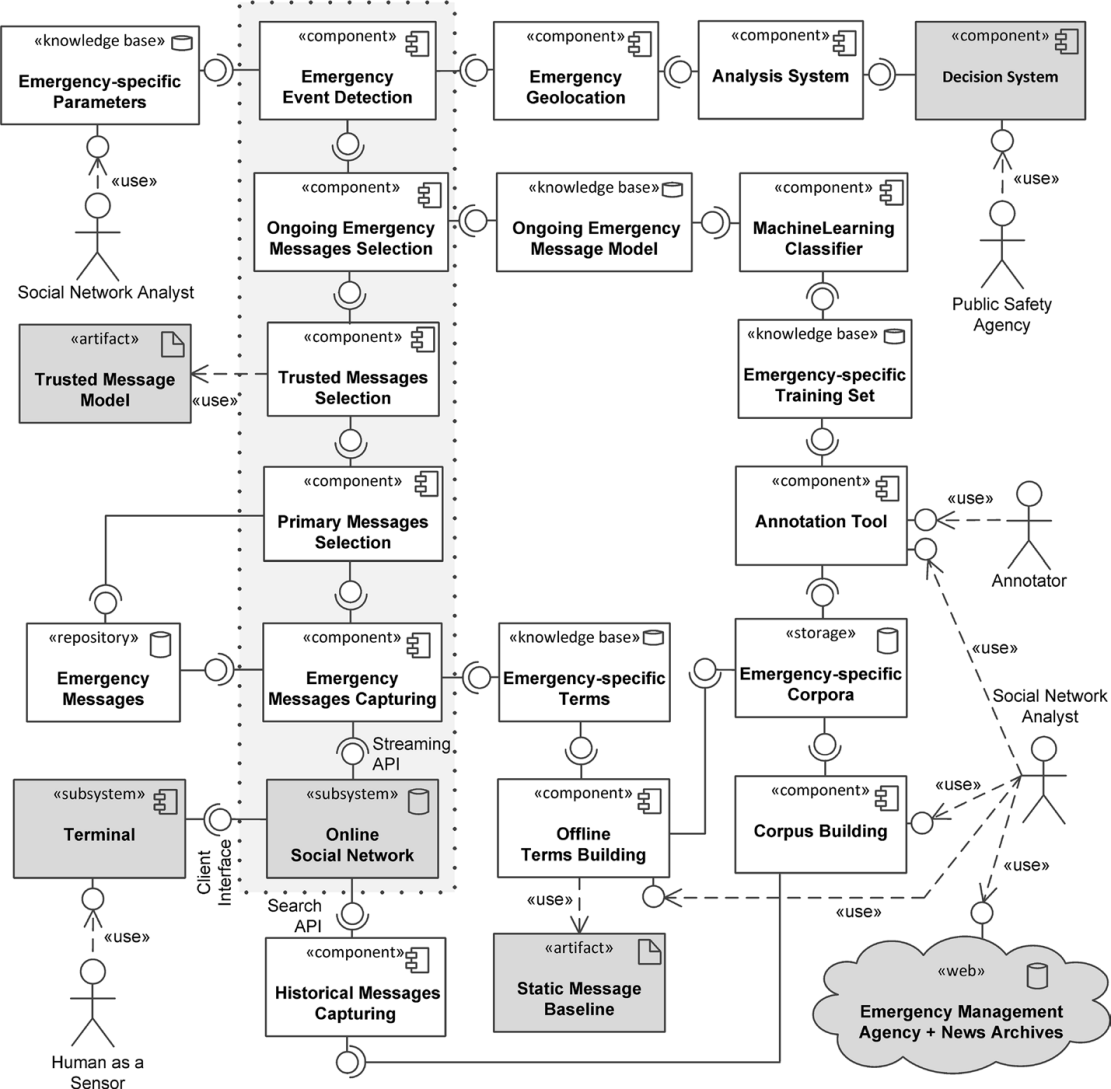
The logical architecture of a decision support system for emergency management based on social sensing

Figure 3 shows a UML static view of the system, made by components and their interfaces. Here, a component is represented by a box, with provided and required interfaces represented by the “lollipop” and “socket” icons, respectively. Actors are represented by the “stick man” icon. Components that are external to the design are colored in dark gray. Some specific types of components or subsystems, such as repository, storage, knowledge base, web, are characterized by a special icon or shape. The usage of a component by an actor or by another component is represented by the socket icon or by the dashed arrow, respectively. The architecture is focused on the social detection system, i.e., on the HaaS input channel. The *Human as a Sensor* actor is represented on the bottom left as an actor using the *Terminal* subsystem to deliver messages to the *Online Social Network* subsystem. The *Online Social Network* subsystem feeds the main data flow carried out in the online mode of operation, i.e., the detection process. In figure, the components involved in the online process are arranged in a stack of components, enclosed in a dotted box, where the *Online Social Network* is a the bottom.

More specifically, the *Emergency Message Capturing* component accesses the *Online Social Network*’s global stream of data, via a streaming API, to collect emergency messages. The messages are captured according to the *Emergency-specific Terms* provided by the knowledge base, and then pushed to the *Emergency Messages* repository, which acts as a buffer with respect to the large data stream provided by the *Online Social Network*. The *Primary Messages Selection* component takes data from this buffer and provides only primary messages to the *Trusted Messages Selection* component, which, in turn, provides only trusted messages to the next component. The semantics of both *primary* and *trusted* is compliant with the HaaS ontology. The latter component employs a statically defined *Trusted Message Model*, which is the same for all types of emergencies. Both components implement fast and coarse-grained filtering to avoid congestion due to the large number of messages.

**Fig. 4:**
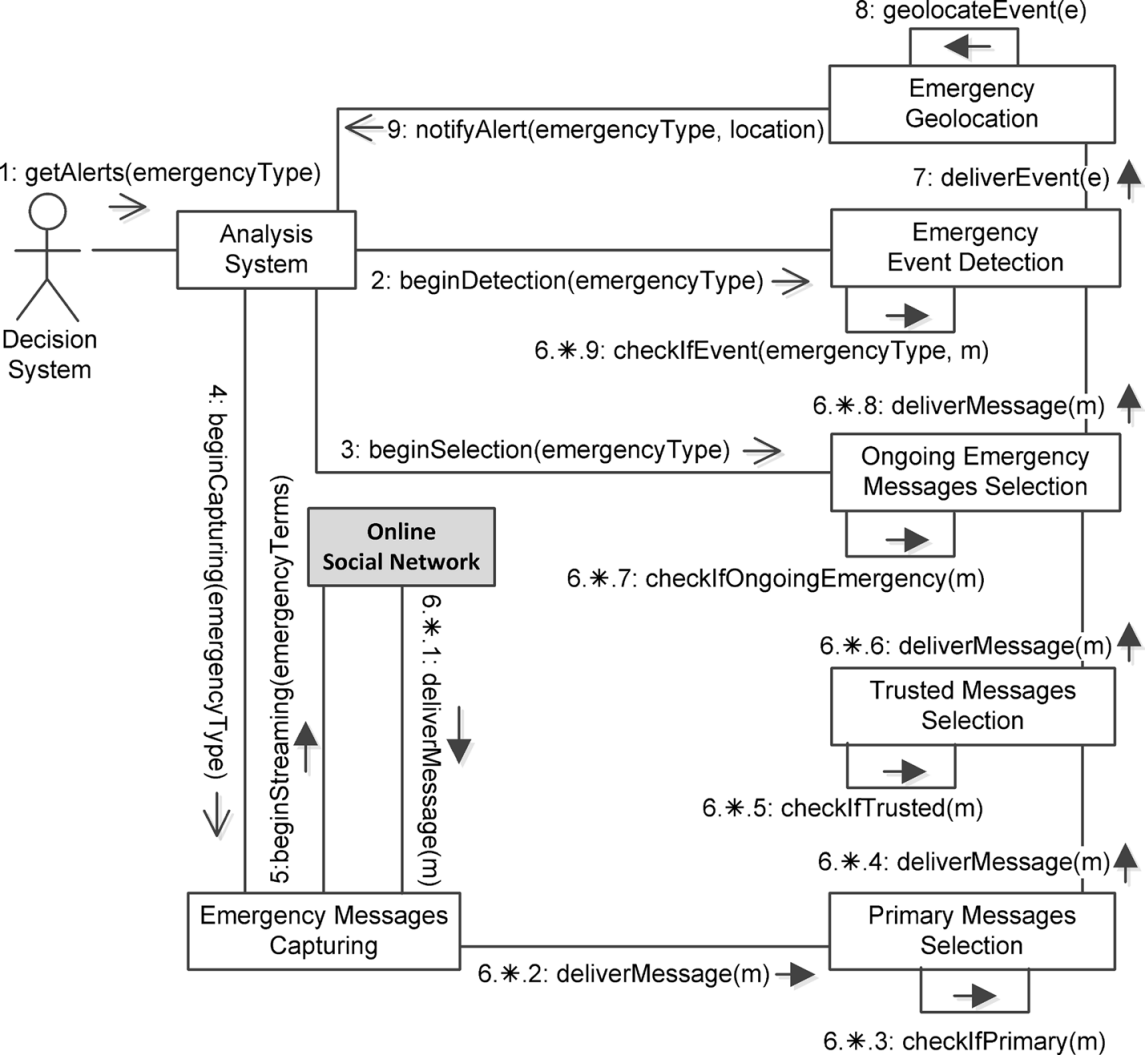
Communication diagram of the online process in a decision support system for emergency management based on social sensing

The next filtering component is the *Ongoing Emergency Messages Selection*, which is fed by the *Trusted Message Selection* component and implements the namesake concept of the HaaS ontology. This component carries out a fine-grained filtering, employing an *Ongoing Emergency Message Model* knowledge base. The outgoing messages are subsequently sent to the *Emergency Event Detection* component, which is able to detect an actual collective emergency. Since each type of emergency needs a different parameterization, this component is based on the *Emergency-specific Parameters* knowledge base configured by the *Social Network Analyst*. The detected event is then gelolocated by the *Emergency Geolocation* component. Finally, the geolocated emergency is provided to the *Analysis System*, which is able to interoperate with a *Decision System* of a *Public Safety Agency*.

In the offline mode of operation, the setting of parametric models and knowledge bases for each type of emergency is covered. This offline process is managed by the *Social Network Analyst* (on the bottom right) with the help of some *Annotators*.

More specifically, given a new type of emergency the web is first accessed to find, via *Emergency Management Agency and News Archives*, some historical examples of the same type of emergency. Subsequently, an *Emergency-specific corpus* of messages is created via the *Corpus Building* component, accessing to the *Online Social Network* via a historical search API managed by the *Historical Messages Capturing* component.

Emergency-specific terms are then created by means of the *Offline Terms Building* component, which uses both the corpus and a *Static Message Baseline* component. A baseline represents common terms in online social networks, which hampers filtering and does not provide relevant information. For this reason, such terms are removed from messages.

Subsequently, an *Emergency-specific Training Set* is created by selecting and annotating messages in the corpus, via an *Annotation Tool*. The training set is finally used to train the *Ongoing Emergency Message Model* via the *Machine Learning Classifier* that exploits a set of features defined on the message corpus itself.

The next subsection provides a dynamic view of the above logical architecture.

### Dynamic view of the logical architecture

In this subsection we focus on the sequence of steps performed by the diverse components in both online and offline processes. Figure 4 shows the online process, via a UML communication diagram. Here, interacting components are connected by temporary links. Messages among components are shown as labeled arrows attached to links. Each message has a sequence number, name and arguments. A message may be asynchronous or synchronous. On an asynchronous call, the execution of the sender continues immediately after the call is issued, and the processing of the message is made by the receiver concurrently with the execution of the sender. On a synchronous call, the execution of the sender is blocked during the execution of the invoked procedure. When the receiver has carried out the procedure, it returns the generated values to the sender, which is awakened and allowed to continue execution. In a communication diagram, synchronous messages are shown with filled arrow head, whereas asynchronous messages have an open arrow head. A return message is denoted by a dashed open arrow head.

**Fig. 5:**
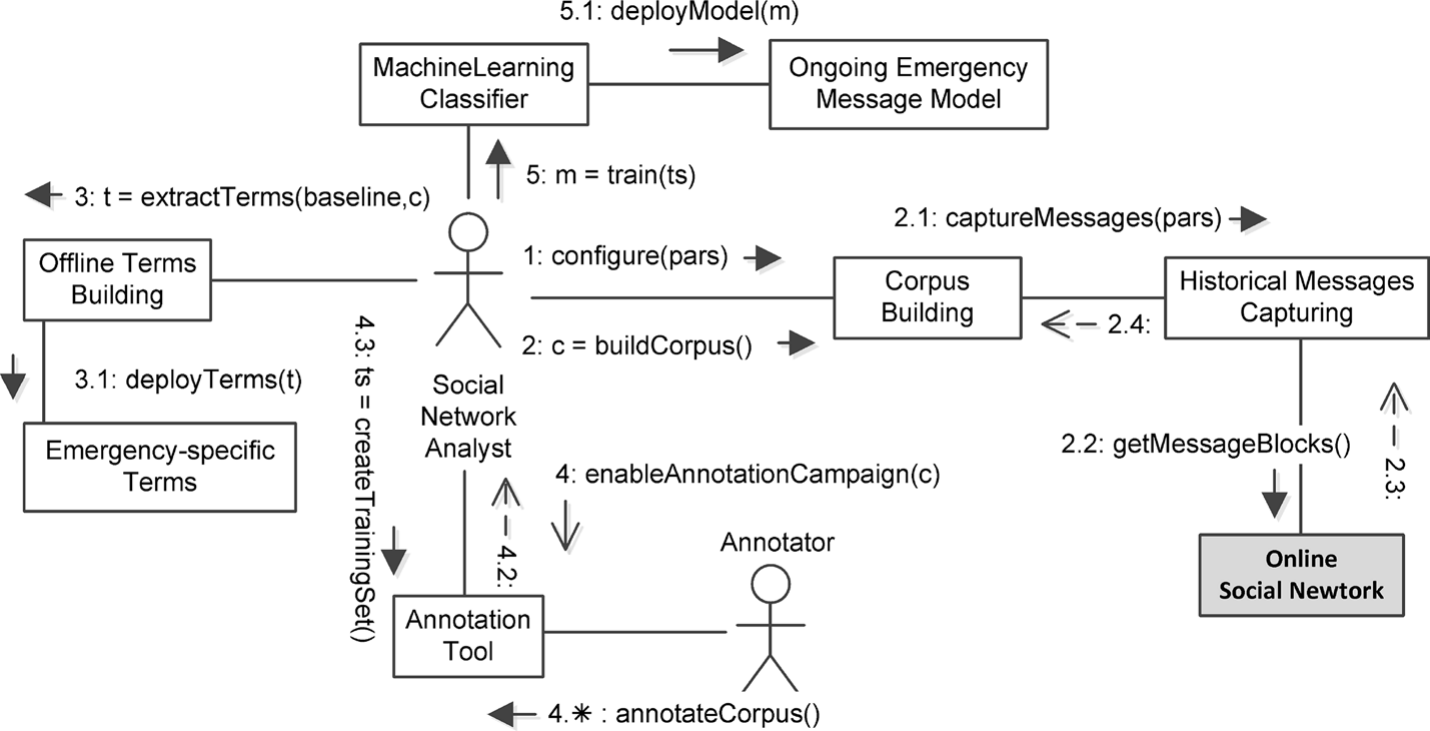
Communication diagram of the offline process in a decision support system for emergency management based on social sensing

Let us suppose that the offline process (as described later in Fig. 5) was previously performed so that the system is ready-to-use for a given type of emergency. The online process evolves as in the following: (1) the *Decision System* makes the *getAlerts* call to the *Analysis System* component, providing the *emergencyType* as a parameter (e.g., “earthquake”, “flooding”); (2–4) the *Analysis System* makes the *beginDetection*, *beginSelection* and *beginCapturing* calls to the *Emergency Event Detection*, *Ongoing Emergency Messages Selection*, and *Emergency Messages Capturing* components, respectively, providing the *emergencyType* as a parameter; (5) the *Emergency Messages Capturing* component makes the *beginStreaming* call to the *Online Social Network* component, providing the *emergengyTerms* as a parameter. The latter call is synchronous, so as to avoid losing data from the *Online Social Network*’s stream. The sixth step is made of a number of substeps iteratively carried out for each message delivered by the *Online Social Network*; for this purpose, the whole step for a given message is referred to as 6.*, whereas the single substep is referred to as 6.*.1, 6.*.2, and so on.

Each emergency message delivered by the *Online Social Network* to the *Emergency Messages Capturing* component (6.*.1), is then delivered to the *Primary Messages Selection* component (6.*.2), which *checks whether the message is primary or not* (6.*.3). If the message is primary, it is delivered to the *Trusted Messages Selection* component (6.*.4), which *checks whether the message is trusted or not* (6.*.5). If the message is trusted, it is delivered to the *Ongoing Emergency Messages Selection* component (6.*.6), which, in turn, *checks whether the message refers to an ongoing emergency or not* (6.*.7). If the message refers to an ongoing emergency, it is delivered to the *Emergency Event Detection* component (6.*.8), which according to an arbitrary detection algorithm (i.e., a message-burst detection algorithm), *checks whether to trigger the detection of an event or not* (6.*.9). When an event occurs, it is received (7) and geolocated (8) by the *Emergency Geolocation* component, and the *Analysis System* is finally *notified with an alert* (9) by the *Emergency Geolocation* component itself.

The offline process, described in Fig. 5, is aimed at providing the *Emergency Messages Capturing* component with *Emergency-specific Terms*, as well as training the *Machine Learning Classifier* component for a new type of emergency. At the beginning, the *Social Network Analyst* is provided with some occurrences of the new type of emergency via historical archives. He needs to build some collection of messages related to such occurrences.

In the first step the *Social Network Analyst configures the Corpus Building* component (1) with some parameters derived from the archives and purposely targeted on each specific occurrence (e.g., date and location of the emergency). Then, the *Social Network Analyst asks the Corpus Building* component to *build the corpus* (2). This is made through two substeps: the *Corpus Building* component asks the *Historical Messages Capturing* component to *capture messages* with the above parameters (2.1), and the *Historical Messages Capturing* component *gets message blocks from the Online Social Network* component (2.2), by using a historical search API. Message blocks are then returned and collected to build the corpus (2.3–2.4).

The *Social Network Analyst*, by using the returned corpus and a baseline of messages from the OSN, asks the *Offline Terms Building* component to *extract Emergency-specific Terms* (3) which are then deployed on a knowledge base (3.1). He also *enables the annotation campaign* of the corpus (4) by enrolling a number of *annotators* (4.*). At the end of the annotation campaign (4.2) the *Social Network Analyst* creates the training set of messages (4.3). The training set is then used by the *Social Network Analyst* to train the *Machine Learning Classifier* component (5) by exploiting the annotated corpus and a set of features defined on the corpus itself. At the end of the training, an *Ongoing Emergency Message Model* is created (5.1).

The model so far created will be used by the *Ongoing Emergency Messages Selection* component during the online process. The *Trusted Messages Selection* and the *Primary Messages Selection* components are ready-to-use for any type of emergencies, and then they do not require training nor setting procedures.

Finally, the *Emergency Messages Capturing* component will employ the *Emergency-specific Terms* created at the third step of the offline process to extract emergency messages from the *Online Social Network* during the online process.

## System implementation

This section describes an implementation of the logical architecture proposed in the previous section, by means of a prototypical application in the domain of *Seismic* emergencies. Such application implements the components involved in the online process (i.e., with reference to Fig. 3, those arranged in a stack on top of *Online Social Network* and enclosed in a dotted, light grey box) to act as a Twitter-based earthquake detector.

### Emergency Messages Capturing

The *Emergency Messages Capturing* component is in charge of gathering messages potentially related to an emergency. As the overall online process relies on data collected at this stage, this component plays a crucial role within the framework. As shown in Fig. 3, *Emergency Messages Capturing* interfaces directly to the *Online Social Networking* platform, provided by Twitter, and exploits the *Emergency-specific Terms* knowledge base, which is generated and updated by the offline process. This knowledge base contains the keywords used by the *Emergency Messages Capturing* component to query the Twitter platform in order to capture earthquake-related messages (e.g., for *Seismic* emergencies in Italy, it contains the two italian terms “terremoto” (earthquake) and “scossa” (tremor)).

Among the methods provided by Twitter for data capturing, the implemented system exploits the Streaming API^5^ to open a persistent connection with a stream of tweets. The Streaming API gives access to a global stream of messages, optionally filtered by search keywords. In contrast with the Search API used in the systems described in Sakaki et al. (2010, 2013), Earle et al. (2012), Yin et al. (2012), Robinson et al. (2013), which gives access only to a subset of all the tweets produced, the Streaming API potentially makes it possible to capture all the tweets matching the search criteria. To guarantee the robustness and the reliability of the system we also implemented additional mechanisms that manage rate-limit and generic connection problems in the use of the Streaming API. Such mechanisms include the adoption of a backup streaming connection to avoid loss of data in case of a sudden disconnection from the primary stream, and mechanisms to perform automatic reconnection upon disconnecting from a stream. Twitter rate-limits for the Streaming API^6^ are set so as to deliver, at any given time, at most 1 % of the total worldwide Twitter traffic, per streaming connection. However, our system never suffered from such a limitation over a 2 months long experiment, during which the collected tweets never generated a traffic exceeding the 1 % threshold. Applications exploiting Twitter’s Streaming API should also guarantee a rapid processing of delivered messages. Clients which are unable to process messages fast enough will be automatically disconnected by Twitter. This situation is commonly refered to as *Falling Behind*. Following Twitter’s guidelines, in our implementation we decoupled the data capturing and analysis phases by rapidly storing messages in a NoSQL MongoDB^7^ database. Such messages are later properly formatted and copied in a relational MySQL database for further processing.

It should be noted that not all the messages gathered in this first step are actually related to an unfolding seismic event. In fact, some messages can be misleading for the event detection task and must be filtered out as noise (Earle et al. 2012). For example, their contents could be maliciously fictitious, convey reported news or talk about past of future events. This motivates the filtering components required by the architecture and described in the following.

### Primary Messages Selection

The *Primary Messages Selection* component is the first filtering module in the proposed architecture and is therefore fed with the whole stream of messages gathered by the *Emergency Messages Capturing* component. Due to the potentially large volume of messages to be processed at this stage, this component performs a fast coarse-grained filtering of incoming messages by applying heuristic rules to select *firsthand* tweets sent by *eyewitness* users who are actually present at the referred event and can directly describe it (Kumar et al. 2013; Morstatter et al. (2014)).

Studying the characteristics of the messages shared on Twitter in the aftermath of seismic events led us to the observation that genuine reports of earthquakes do not follow any information diffusion model and are not influenced by other reports. However, this scenario rapidly evolves over time as the news of the earthquake spreads over the different medias, so that subsequent reports are in growing percentage influenced by other news. Thus, we concluded that the best results for the event detection task could be achieved by considering only spontaneous and independent messages. The *Primary Messages Selection* component therefore discards retweet messages, reply messages and messages shared by accounts belonging to a blacklist of 345 Twitter profiles that publish official information about recent emergencies. We are aware that the heuristics exploited by the *Primary Messages Selection* component might not be enough to discard all derivative messages. Nonetheless, they represent a computationally efficient way of filtering out the vast majority of useless messages. Furthermore, the modular and architectural solution we propose is particularly suitable for being expanded with alternative approaches and algorithmic solutions to this task.

**Fig. 6:**
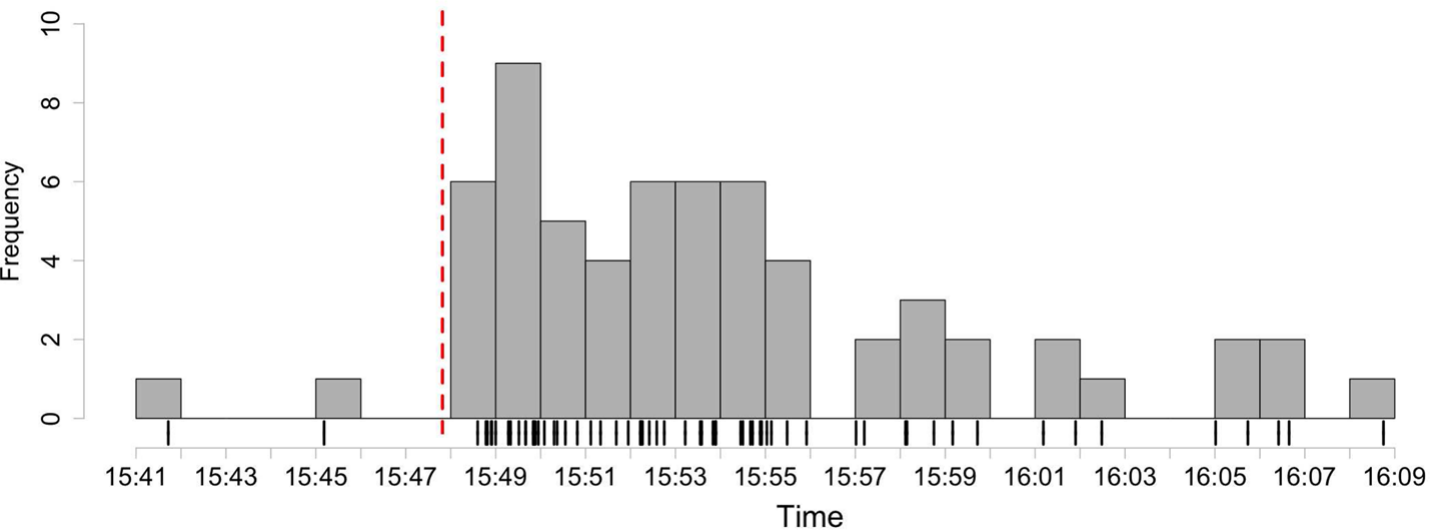
A burst of messages registered after a moderate earthquake

### Trusted Messages Selection

Another possible flaw for all social mining systems lies in the vulnerability to intentional attacks performed by malicious users (Mendoza et al. 2010; Castillo et al. 2011). In our application, security concerns can arise if groups of people collude to generate fictitious tweets referring to an earthquake. The online *Trusted Messages Selection* component exploits the *Trusted Message Model* to select trusted, reliable messages. Many already developed classifiers can be exploited for this task, such as the ones proposed in Chu et al. (2012) and Amleshwaram et al. (2013). In our implementation we employ a domain-independent machine learning classifier trained to distinguish between “fake” and “real” accounts (Cresci et al. 2014, 2015a). The classifier has been trained on a set of 3900 equally distributed fake and real accounts and was able to correctly classify more than 95 % of the accounts of the training set. In the online mode of operation, the *Trusted Messages Selection* component exploits the trained model and the Weka tool (Hall et al. 2009) to infer the class (fake, real) a user who posted a message belongs to. The *Trusted Messages Selection* component performs this operation for every message it receives from the *Primary Messages Selection* component. Messages posted by fake users are automatically discarded by the system. In addition, users repeatedly triggering false detections are added to the same account blacklist exploited by the *Primary Messages Selection* component. To further protect the system from harmful attacks, we consider only a single message per user, and messages from different users but with the same contents are considered only once. While we understand that these solutions do not fully address the problem of malicious attacks, we are confident that our efforts represent a first response to security concerns in social mining systems. In fact, the adopted solutions require potential attackers to put considerably much effort into the creation of plausible accounts. The employment of the solutions proposed in Chu et al. (2012) and Amleshwaram et al. (2013) for the classification of “automated” versus “non-automated” accounts, might represent another possible way to tackling this problem and stands as promising ground for future work.

### Ongoing Emergency Messages Selection

To further enforce the *Primary*, *Trusted* and *Emergency* message properties, the *Ongoing Emergency Messages Selection* component performs a fine-grained filtering by means of the *Ongoing Emergency Message Model*, a machine learning classifier which has been trained in the offline process. Again, we exploited Weka to train and generate the classifier. The *Emergency-specific Training Set* for earthquakes is composed of more than 1400 tweets divided into two balanced sets of messages: tweets related and tweets not related to a seismic event in progress. During the offline phase, tweets of the training set were manually classified by the *Annotators* using the ad-hoc *Annotation Tool* web interface^8^. Our analysis of the messages reporting earthquakes has highlighted a few interesting characteristics that help distinguish between tweets related and tweets not related to an unfolding seismic event. Tweets referring to an earthquake are generally very short, they present fewer punctuation than normal tweets and often contain slang or offensive words. This is because people reporting an earthquake are usually scared about the event and the content of the messages they write tend to reflect this emotion. Instead, tweets referring to official news of an earthquake or talking about a past earthquake present a longer, more structured message. Tweets not related to a recent earthquake also include a higher number of mentions and URLs than spontaneous earthquake reports. Thus, we defined the following set of features that takes into account the results of the previous analysis: (1) character count; (2) word count; (3) punctuation count; (4) URL count; (5) mention count; (6) slang/offensive word count. Notably, some of the features that we defined for this task are also supported by the findings of recent related works (Imran et al. 2013; Gupta et al. 2013).

Training the classifier with this set of features produced correct classifications in more than 90 % of the tweets of the *Emergency-specific Training Set*. The classifier was obtained using the decision tree J48, corresponding to the Java implementation of the C4.5 algorithm (Quinlan 1993) with a tenfold cross validation. In the online mode of operation, the prediction is performed by invoking the classifier every time a message is delivered to the *Ongoing Emergency Messages Selection* component. As Weka generally needs less than a second to predict the class of a new tweet by means of our decision tree model, it is feasible to use the fine-grained classifier filter at this stage of the system since most of the noisy messages have already been filtered out by previous components.

### Emergency event detection

The detection of a seismic event is triggered by an exceptional growth in the frequency of messages that have passed the filtering phases. In our system, we adopt a novel event detection approach which is based on a burst detection algorithm. A burst is defined as a large number of occurrences of a phenomenon within a short time window (Zhang and Shasha 2006). Burst detection techniques are commonly applied to various fields such as the detection of topics in data streams. Our system triggers the detection of a seismic event when it identifies a burst of *Ongoing Emergency Messages*. Figure 6 displays a rug plot of the arrival times of *Ongoing Emergency Messages*, as well as a histogram plot showing their frequency per minute, during a 3.4 magnitude earthquake occurred at 15:47:49, August 9 2014, in Tuscany regional district. After the occurrence time of the earthquake, denoted by the red vertical dashed line, a big burst of tweets was recorded by our system.

**Table 1:** Earthquake detection validation

Magnitude	Earthquakes	Detection results			Validation metrics		
		TP	FP	FN	Precision (%)	Recall (%)	F-Measure (%)
*System validation against all the earthquakes registered by INGV*							
>2.0	404	17	30	387	36.17	4.21	7.54
>2.5	102	16	30	86	34.78	15.69	21.62
>3.0	26	13	17	13	43.33	50.00	46.43
>3.5	11	9	3	2	75.00	81.82	78.26
>4.0	7	5	0	2	*100*	71.43	83.33
>4.5	2	2	0	0	*100*	*100*	*100*
*System validation against earthquakes that generated at least one report on Twitter*							
>2.0	128	17	30	111	36.17	13.28	19.43
>2.5	55	16	30	39	34.78	29.09	31.68
>3.0	21	13	17	8	43.33	61.90	50.98
>3.5	9	9	3	0	75.00	*100*	85.71
>4.0	5	5	0	0	*100*	*100*	*100*
>4.5	2	2	0	0	*100*	*100*	*100*

Excellent values for the validation metrics are reported in italics

Works in Kleinberg (2003), Ebina et al. (2011) discuss various burst detection algorithms. Our *Emergency Event Detection* component implements the hierarchical algorithm proposed in Ebina et al. (2011) since it is computationally light and can adapt well to both big and small bursts. An efficient algorithm is necessary because of the real-time nature of our system, and the ability to detect both big and small bursts fits well with the need of a flexible, scalable and reusable system.

## Experimental studies

The validation of the proposed *Social Detection System* has been carried out exploiting official data released by the National Institute of Geophysics and Volcanology^9^ (INGV), the authority responsible for monitoring seismic events in Italy. INGV uses different channels, including a dedicated Twitter account^10^, to distribute detailed information about seismic events having magnitude 2 or more, which have been detected by their seismographic network. To validate the proposed architecture, we cross-checked all the events detected by the prototypical application described in the previous section, against the official reports released by INGV. This approach allowed us to validate our system with stronger metrics than the ones used in similar works, such as Sakaki et al. (2010, 2013), Earle et al. (2012) and Yin et al. (2012), Robinson et al. (2013). Specifically, the majority of social media emergency management systems have been validated with a focus on correct detections. However, the problem of false detections is often understated, despite being a critical factor in emergency management (Middleton et al. 2014). Therefore, we classified earthquake detection results as in the following:

- *True Positives (TP)* events detected by our system and confirmed by INGV;
- *False Positives (FP)* events detected by our system, but not confirmed by INGV;
- *False Negatives (FN)* events reported by INGV but not detected by our system.

True Negatives (TN) are widely used in information retrieval and classification tasks together with TP, FP and FN. However, in our scenario TN are not applicable, as it would mean counting the number of earthquakes that did not happen and that our system did not detect. In addition, we also computed the following standard metric

- *Precision*, ratio of correctly detected events among the total number of detected events:
  $$\textit{Precision}=\frac{TP}{TP+FP}$$
- *Recall (a.k.a. Sensitivity)*, ratio of correctly detected events among the total number of occurred events:
  $$\textit{Recall}=\frac{TP}{TP+FN}$$
- *F-Measure*, harmonic mean of Precision and Recall:
  $$\textit{F-Measure}=2*\frac{Precision*Recall}{Precision+Recall}$$

We were not able to compute other well-known metrics such as Specificity, Accuracy and Mathews Correlation Coefficient since they rely on the True Negatives (TN) count. Employed metrics are anyway exhaustive and allow a thorough validation of detection results. Table 1 summarizes event detection validation against earthquakes registered by INGV over a 66 days time window starting from 2013-07-19 to 2013-09-23. The number of earthquakes reported in Table 1 refers only to real earthquakes detected by INGV and therefore corresponds to the sum of TP and FN. FP instead represent false detections by our system.

We first evaluated the *Social Detection System* against all the earthquakes having a magnitude greater than 2.0, registered by INGV within the given time window. Results show that the detection of earthquakes with magnitude lower than 3 is a very challenging task. This is because the majority of these earthquakes are only detected by seismographic stations and not by people. For events with a magnitude equal to or greater than 3.5, results show a good performance of the system, as demonstrated by the encouraging values of F-Measure: 78.26 % for magnitude >3.5, 83.33 % for magnitude >4 and 100 % for magnitude >4.5. This is especially significant given that seismic events of a magnitude around 3 are considered “light” earthquakes and are generally perceived only by a very small number of social sensors.

The majority (68 %) of the earthquakes occurred during the 66 days validation time window were extremely light and did not generate any report on Twitter. A detection system based solely on tweets is obviously incapable of detecting such events and this is reflected by the high number of False Negatives (FN) and by the low Recall for earthquakes with magnitude lower than 3.

In the emergency management scenario, light seismic events only detected by seismographic stations clearly do not pose any threat to communities and infrastructures and earthquakes of interest are those actually felt by the population at large. Therefore we re-validated the system against those earthquakes that generated at least one report on Twitter. Results for this experiment are displayed in the bottom half of Table 1 and show an overall improvement in the system performances. It is worth noting that the proposed *Social Detection System* achieves flawless results (Precision, Recall and F-Measure = 100 %) for earthquakes of magnitude 4.0 or more and still performs very well on earthquakes which have a magnitude in the region of 3.5 (Precision = 75 %, Recall = 100 % and F-Measure = 85.71 %).

**Fig. 7:**
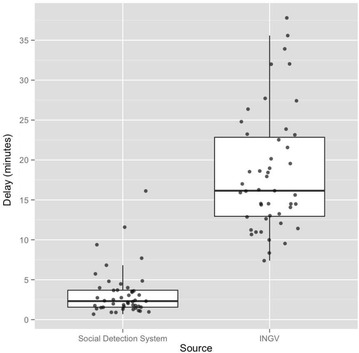
System responsiveness validation. Distribution of detection delays versus INGV notification delays

Figure 7 characterizes the system’s responsiveness by means of boxplot and scatterplot distributions of the detection delays of our system compared to the notification delays of INGV official reports. The detection delays of our *Social Detection System* are computed as the difference between the occurrence timestamp of an earthquake and the timestamp of the corresponding detection triggered by the *Emergency Event Detection* component. INGV notification delays are computed as the difference between the occurrence timestamp of an earthquake and the timestamp of the corresponding official report released by INGV. The detection delays reported in Fig. 7 have been computed considering only True Positive detections.

INGV official reports are the timeliest publicly available source of information about earthquakes in Italy. Anyway, INGV notification delays are considerably higher than the detection delays of our system. In Fig. 7 this is evident from the massive gap between the spreads (or boxes) of the two distributions. Earthquake detection responsiveness of our system is even more valuable since early reports of severe earthquakes might be of interest not only to emergency responders, but also to all breaking news agencies looking for fresh information to publish as well as to insurance companies and financial advisors.

Among all the detections performed by our system, 87 % occurred within 5 minutes of the earthquake and 43 % occurred within 2 minutes. These results are promising, especially considering that the proposed framework is adaptable to other emergency scenarios where automatic detection equipment, playing the role of seismographs for seismic events, might not be available. Being able to automatically detect a considerable percentage of emergency situations within minutes of the event would surely benefit emergency responders.

## Conclusions and future work

In this paper we have discussed how the HaaS paradigm can be exploited for emergency detection. Core concepts, major roles and functionalities have been specified to operate in a broad class of emergencies. The design of architectural components reusable for many types of events, and possibly adaptive with respect to the different characteristics of each type, has been detailed. Related works have been discussed via the proposed architectural model, to systematize the available solutions under our modular and platform-independent conceptual framework. The implementation of an actual Twitter-based earthquake detector has been then presented, to show the effectiveness of our approach. Furthermore, a real-world case of application has been discussed and analyzed, discovering the most interesting properties of our approach. In addition, the architecture has been validated under more comprehensive metrics with respect to the existing literature.

As a future work, to better assess the system over its whole life cycle, it should be cross-validated on other real-world scenarios, involving emergencies of different types and sizes. Afterwards, the next key investigation activities along this line of research should be to employ real-time data provided by bursts of messages as a mine of information for situational awareness and damage assessment. Specifically, qualitative analyses of relevant messages can be performed to increase the overall situational awareness in the aftermath of an emergency. Qualitative analyses of the textual content of messages can be performed via natural language processing techniques and might lead to time-evolving term-clouds, highlighting those textual bits which convey critical and actionable information. In parallel, analyses of the multimedia content of messages can be carried out by means of image filtering and image clustering techniques. However, despite providing valuable insights into the unfolding scenario, the output of qualitative analyses still requires to be interpreted by domain-experts. In contrast, quantitative analyses could provide unambiguous outputs which might prove even more valuable to decision-makers and emergency responders. Specifically, for seismic events, a quantitative approach to the estimation of the impact of an earthquake can be performed by training statistical regression models to estimate earthquake intensity from the characteristics of social media reports.

In the future we look forward to addressing these issues by extending our modular framework to include components performing analyses aimed at increasing situational awareness and capable of providing early damage assessments.

## Footnotes

1 http://earthquake.usgs.gov/learn/topics/mag_vs_int.php.

2 https://dev.twitter.com/rest/reference/get/search/tweets.

3 http://www.livescience.com/45385-earthquake-alerts-from-twitter.html.

4 https://blog.twitter.com/2015/usgs-twitter-data-earthquake-detection.

5 https://dev.twitter.com/streaming/overview.

6 https://dev.twitter.com/streaming/overview/messages-types#limit_notices.

7 http://www.mongodb.org/.

8 http://wafi.iit.cnr.it/sosnlp/sosnlp/annotation_tool.

9 http://www.ingv.it/en/.

10 https://twitter.com/ingvterremoti.
